# Invasion characteristics of a *Plasmodium knowlesi* line newly isolated from a human

**DOI:** 10.1038/srep24623

**Published:** 2016-04-21

**Authors:** Amirah Amir, Bruce Russell, Jonathan Wee Kent Liew, Robert W. Moon, Mun Yik Fong, Indra Vythilingam, Vellayan Subramaniam, Georges Snounou, Yee Ling Lau

**Affiliations:** 1 Department of Parasitology, Faculty of Medicine, University of Malaya, Kuala Lumpur 50603, Malaysia; 2 Department of Microbiology, Yong Loo Lin School of Medicine, National University of Singapore, National University Health System, Singapore 117545, Singapore; 3 Department of Immunology and Infection, Faculty of Infectious and Tropical Diseases, London School of Hygiene and Tropical Medicine, London, WC1E 7HT, United Kingdom; 4 Department of Pharmacology and Chemistry, Faculty of Pharmacy, Universiti Teknologi MARA, Puncak Alam Campus, Bandar Puncak Alam, Selangor 42300, Malaysia; 5 Sorbonne Universités, UPMC Univ Paris 06, Inserm (Institut National de la Santé et de la Recherche Medicale), Centre d’Immunologie et des Maladies Infectieuses (Cimi-Paris), UMR 1135, ERL CNRS 8255 (Centre National de la Recherche Scientifique), 91 Boulevard de l’Hôpital, F-75013 Paris, France

## Abstract

*Plasmodium knowlesi* is extensively used as an important malaria model and is now recognized as an important cause of human malaria in Malaysia. The strains of *P. knowlesi* currently used for research were isolated many decades ago, raising concerns that they might no longer be representative of contemporary parasite populations. We derived a new *P. knowlesi* line (University Malaya line, UM01), from a patient admitted in Kuala Lumpur, Malaysia, and compared it with a human-adapted laboratory line (A1-H.1) derived from the *P. knowlesi* H strain. The UM01 and A1-H.1 lines readily invade human and macaque (*Macaca fascicularis*) normocytes with a preference for reticulocytes. Whereas invasion of human red blood cells was dependent on the presence of the Duffy antigen/receptor for chemokines (DARC) for both parasite lines, this was not the case for macaque red blood cells. Nonetheless, differences in invasion efficiency, gametocyte production and the length of the asexual cycle were noted between the two lines. It would be judicious to isolate and characterise numerous *P. knowlesi* lines for use in future experimental investigations of this zoonotic species.

*Plasmodium knowlesi* was first officially described in India in the early 1930’s in a *Macaca fascicularis* specimen from Singapore^1^. Whereas it causes mild and chronic infection in its natural hosts (*M. fascicularis* and *M. nemestrina*), *P. knowlesi* infections in rhesus macaques (*M. mulatta*) run a fulminant course and are usually rapidly lethal if untreated. The ease with which this parasite can be maintained and transmitted in the laboratory made it a favoured model for numerous immunological, physiological and chemotherapeutic investigations. Over the years, other strains were isolated from animals or anophelines in Malaysia and neighbouring countries^2^ and some were used for malaria research^3^.

Soon after the initial isolation of *P. knowlesi*, humans were found to be susceptible to experimental infections by *P. knowlesi* that in some led to severe symptoms^4^,^5^,^6^. The first confirmed natural infection in humans was only recorded thirty years later, thus providing the first proof of a zoonotic malaria infection in humans. The infecting line (H strain) from this case was isolated^7^ and is still employed for scientific investigation. In recent years a focus of *P. knowlesi* infections was discovered in Sarawak^8^. At present this species is the most important cause of malaria in residents of Peninsular Malaysia, Sarawak and Sabah, with cases occasionally recorded from the neighbouring countries where the natural simian hosts occur^9^.

The confirmed zoonotic potential of *P. knowlesi* has re-enforced the value of this species for fundamental research on the biology of malaria parasites. Most notably, the phenomenon of antigenic variation in malaria was first uncovered using *P. knowlesi*^10^, and the seminal studies on the invasion of red blood cells by merozoites were based on *P. knowlesi*^11^ and led to the first demonstration of an absolute requirement for the Duffy receptor for erythrocyte invasion by a malaria parasite^12^.

With the exception of some recent *ex vivo* drug sensitivity and cytoadherence assays using field isolates, the bulk of the investigations carried out using *P. knowlesi* employed strains that had been principally maintained by blood passages in *M. mulatta* for half a century or more. More recently, the amenability of *P. knowlesi* to genetic manipulation^13^ has prompted successful efforts to adapt the H strain to long-term continuous culture in human red blood cell from which cloned lines were derived^14^,^15^. Such long periods of propagation in cells from the non-natural hosts might have altered the characteristics of the parasite. In this report, we have isolated a *P. knowlesi* line (UM01 line) from a human patient who had acquired the infection recently, and then expanded it in captive non-immune *M. fascicularis*, the natural host. This line was then compared for some of its invasive characteristics to those of one of the H strain’s cloned lines (A1-H.1) that were adapted to human red blood cells^15^.

## Results

### Novel *P. knowlesi* strain isolation

**Figure 1:**
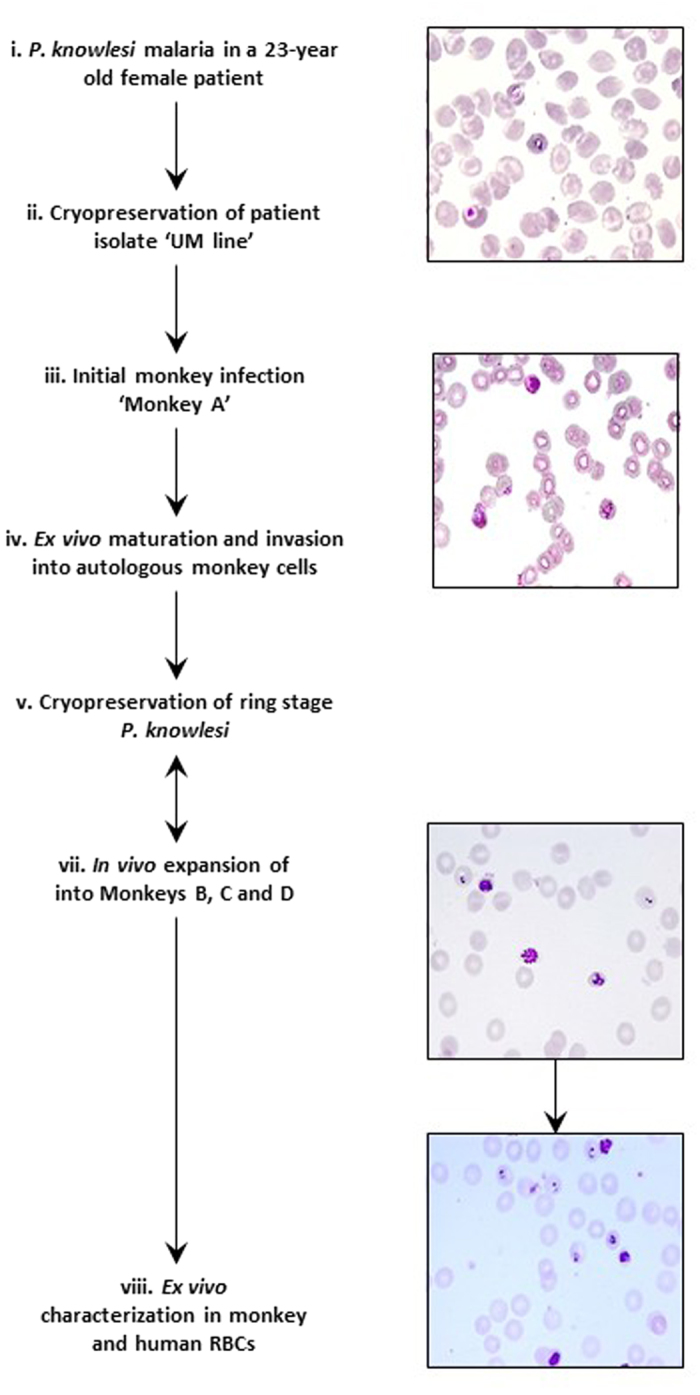
Isolation, expansion of the *P. knowlesi*, UM01 line. The UM01 line was isolated from a knowlesi malaria patient and expanded by passaging it through *M. fascicularis* (macaque A, B, C and D). The ring stages of the UM01 line obtained from the expansion were cryopreserved until further use. Parasites obtained either from *in vivo* or *ex vivo* maturation were used for invasion and inhibition experiments.

In 2013, a 23-year old female presented to University Malaya Medical Centre with a fever history of six days. The infection was probably acquired in a forested area in Hulu Langat District, Selangor, Malaysia a few weeks prior to admission. Blood films revealed a *P. knowlesi* 0.25% parasitaemia that was later confirmed by PCR (Singh *et al.*^8^). An admission blood sample was collected for cryopreservation as line UM01. One month later, a stabilate was thawed before being inoculated into a malaria naive *M. fascicularis* (macaque A, Fig. 1). Eight days post-inoculation when the parasitaemia was 2.6% (mainly late trophozoite stage parasites) 2 mL of whole blood were incubated *ex vivo* for 15 hours during which the parasites matured to schizonts, and then released invasive merozoites yielding a two-fold increase in the parasitaemia. The resulting predominantly ring stage parasites were then cryopreserved, and one of the stabilates from this first passage was thawed later to infect three malaria-naive *M. fascicularis* (macaque B, C and D). Over a ten-day post inoculation period, ten 1 mL aliquots (*P. knowlesi* parasitaemia of 2–15%) were collected from each macaque and were either cryopreserved or immediately used in *ex vivo* invasion assays (Fig. 1). The full asexual development of the UM01 line was consistently 24 hours (+/− 1 hour) under *ex vivo* maturation conditions matching those observed *in vivo* in the macaque.

### *Ex vivo* invasion assays

#### Red blood cell tropism and species specificity

**Figure 2:**
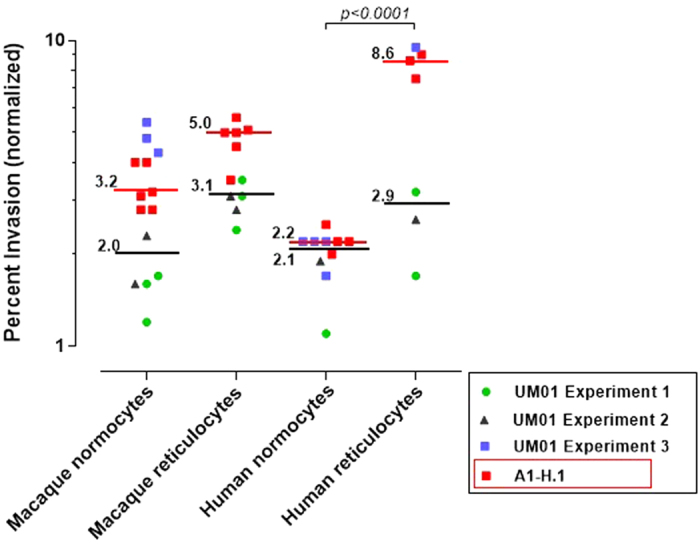
Invasion studies with *P. knowlesi.* *P. knowlesi* (UM01 and A1-H.1 strains) invasion in macaque and human normocytes and reticulocytes; bars = median values (black for the UM01 line and red for the A1-H.1 line). The effect of red blood cell species (human *vs* macaque) and age (normocyte *vs* reticulocyte) was compared using a 1Way ANOVA and Tukey’s Multiple Comparison Tests.

We wished to determine whether the UM01 line displayed any preference to invade *M. fascicularis* or human red blood cells. We also aimed to characterize any tropism towards the red blood cell types for these hosts, using the A1-H.1 line as a comparator. Three independently conducted *ex vivo* assays revealed that the UM01 and the A1-H.1 lines invade both normocytes and reticulocytes, with a preference for reticulocytes in both host species (Fig. 2 and supplementary Table S1). Macaque and human normocytes were invaded to a similar extend by both *P. knowlesi* lines (Fig. 2 and supplementary Table S1).

**Figure 3:**
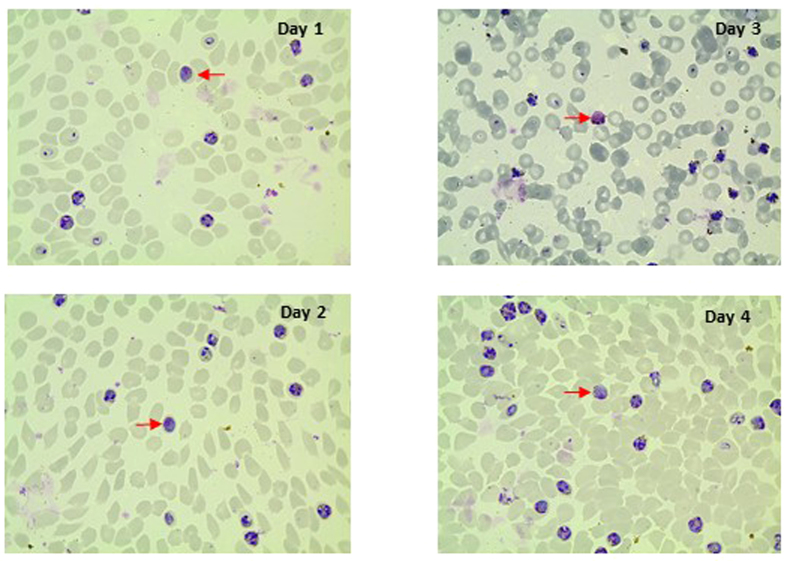
Giemsa stained blood smears of UM01. Gametocytes (red arrow) were observed at different days of culture.

During the course of these experiments, gametocytes were readily observed in all *ex vivo* experiments involving the UM01 line, but in none where the A1-H.1 line was used. As *P. knowlesi* undergoes the classic “short” gametogenesis observed in most malaria parasite species (with the exception of *P. falciparum*) only the relatively mature forms can be readily identified. Short-term culture of the UM01 line demonstrated a gametocyte conversion rate of 2.0 ± 2.4 (Table 1). Due to variations in staining intensity between slides we were unable to discriminate between male and female gametocytes with sufficient confidence (Fig. 3).

#### Invasion inhibition assays

**Figure 4:**
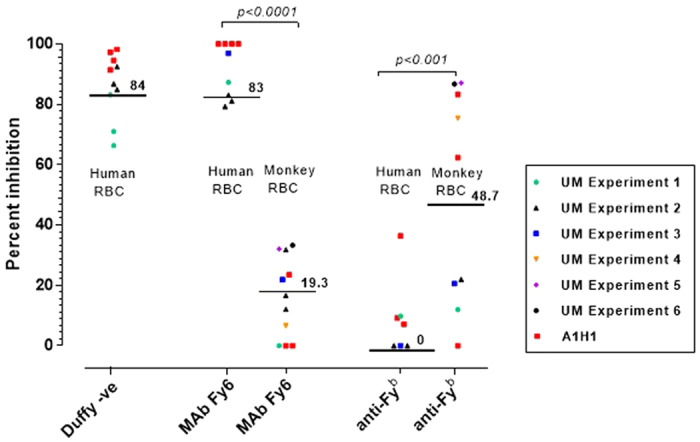
Characterising the DARC dependence of *P. knowlesi* merozoites for the invasion of human and macaque normocytes. Inhibition of *P. knowlesi* (UM01 and A1-H.1 lines) invasion into human (Hu) and macaque (Mc) normocytes by MAb Fy6 and anti-Fy^b^ (Duffy negative human blood was used as a positive control). Bars show the percentage grand median inhibition levels normalised to the Ab-free control of each independent experiment for the UM01 line. The effect of MAb Fy6 and anti-Fy^b^ in invasion inhibition in both human and macaque blood was compared using a 1Way ANOVA and Tukey’s Multiple Comparison Tests.

The Duffy antigen/receptor for chemokines (DARC) dependence of *P. knowlesi* strains, such as the H strain, has been well characterized in humans and *M. mulatta*^16^,^17^. Thus, we sought to characterize the DARC dependence of the new *P. knowlesi* UM01 line for both human and *M. fascicularis* normocytes. DARC negative red blood cells (collected from African donors at University Malaya) confirmed the DARC-dependence of the invasion of human normocytes by both the A1-H.1 line and the UM01 line, though some variability was noted for the UM01 line. Given that DARC-negative *M. fascicularis* blood is not available we used two antibodies that target different amino acids of the DARC N-terminal region (anti-Fy^b^ and MAb Fy6)^18^. As expected, the antibody targeting the Fy6 region completely abrogated the invasion of human normocytes by the A1-H.1 line and substantially so for the UM01 line (Fig. 4 and supplementary Table S2). However, the anti-Fy6 antibody led to only minor inhibition of macaque normocytes invasion (Fig. 4) by both the UM01 line and the A1-H.1 line. The inhibition afforded by the anti-Fy^b^ antibody was low for the invasion of human red blood cells by the two lines, and highly variable for that of macaque red blood cells by both parasite lines (Fig. 4).

## Discussion

Under experimental conditions it is quite common for a given *Plasmodium* species to infect host species other than the natural hosts. Transfer between vertebrate host species often results in modifications of the characteristic course of the infection. Similar changes have also been recorded for parasites experimentally maintained by blood-to-blood passage or in splenectomised hosts. These alterations (which can concern morphology, cytoadhesion, virulence, transmissibility etc.…) underline the biological plasticity of malaria parasites. The basis for such phenotypic variability remains largely unknown and could be due to host or parasite factors or both, including red blood cells properties or altered expression of parasite proteins.

The isolation of a new *P. knowlesi* line from a patient provided an opportunity to compare its invasion characteristics with those of a cloned line adapted to cultivation in human red blood cells that was similarly obtained in 1963. Side-by-side comparisons in the invasion assays of human and *M. fascicularis* red blood cells indicated that the two lines did not differ significantly in efficiency or tropism. In either case, the preference for reticulocytes was far less strict than that observed for *P. vivax* with respect to human reticulocytes^19^. The UM01 and A1-H.1 lines were clearly highly dependent on the presence of DARC on human red blood cells, with some variability observed for the UM01 line. The data from the anti-DARC monoclonal antibodies do not allow a clear-cut interpretation. Macaque species, along with many other non-human primate species, are Fy^a^ negative with a variable Fy^b^ phenotype^20^. This probably accounts for the high variability in the invasion inhibition of the macaque red blood cells in the presence of the anti-Fy^b^ antibody (Fig. 4). Thus, it is likely that *P. knowlesi* is capable of invading its macaque host red blood cells via a DARC-independent pathway, confirming previous observations^21^,^22^,^23^,^24^.

Two differences were noted during the observations made with the two lines. First, the duration of the erythrocytic cycle for the cloned UM01 line was consistently shorter than that of the A1-H.1 line, and second, gametocyte production appeared to be impaired in the A1-H.1 line. It is likely that these differences are due to the long periods of *in vitro* cultivation that were needed to adapt the A1-H.1 line to human red blood cells. Interpretation of our observations should take into account the possibility that the UM01 line might not be clonal and might contain more than one parasite genotype.

The observations presented here can only be taken as preliminary indications of the potential phenotypic diversity of *P. knowlesi* parasites. This species is distributed throughout Southeast Asian countries in geographically isolated regions, some of which are islands. The differences noted for the various isolates prompted malariologists to class some of these as distinct subspecies^2^. This notion is supported by recent molecular analyses of parasites from Borneo, where two genetically distinct populations^25^,^26^,^27^ were identified. Thus, it would be important to establish and characterise *P. knowlesi* lines from each of the geographical areas where this parasite occurs in order to ensure the relevance of future comparative analyses aimed at elucidating biological or pathophysiological mechanisms. Ultimately, reliance on one or two strains of *P. knowlesi* that have been passaged through a multitude of animals over the last 50 to 80 years might significantly limit our understanding of the contemporary populations of *P. knowlesi* that threaten human health today.

## Methods

### Ethics statement

Ethical clearance for the experimental protocols used in this study for humans and macaques blood were obtained and approved by the University of Malaya Medical Centre Medical Ethics Committee (MEC Reference Number: 817.18) and the Institutional Animal Care and Use Committee University of Malaya (Ethics Reference Number: PAR/19/02/2013/AA(R) and PAR/6/03/2015/AA(R)) respectively. All experiments using humans and macaques sample were carried out in accordance with the approved guidelines and regulations. Written informed consent were obtained from all human subjects.

### Sample collection

Blood samples from patients admitted to University Malaya Medical Centre suspected of having malaria were sent in 5 mL lithium-heparinised tubes to “Parasites: South East Asian Diagnostic” (Para:SEAD) laboratory for malaria diagnosis. Diagnosis of *P. knowlesi* infection and parasitaemias were determined by microscopic examination of Giemsa-stained blood films and confirmed by *Plasmodium* species-specific nested-PCR assays^8^. After plasma removal, platelets and leucocytes were removed from *P. knowlesi* positive isolates using a CF11 (Whatman) column^28^ before cryopreserving the filtrate.

### DNA extraction and nested PCR assay

DNA was extracted from 100 μL of patient’s whole blood sample using DNeasy Blood & Tissue Kit (QIAGEN, Valencia, CA, USA) as described by manufacturers. Nested PCR was performed on the extracted DNA to amplify species-specific sequences of the small subunit of the ribosomal RNA (18S SSU rRNA) of *Plasmodium* sp. using primers developed previously^8^,^29^. In the first nested PCR reaction, 5 pmoles of genus specific primers were used (rPLU1: 5′-TCA AAG ATT AAG CCA TGC AAG TGA-3′and rPLU5: 5′-CCT GTT GTT GCC TTA AAC TCC-3′). A volume of 21 μL of PCR mixture (0.25 M dNTP, 1 unit *Taq* polymerase, 1 × PCR buffer (35 mM Tris–HCl [pH 9.0], 3.5 mM MgCl_2_, 25 mM KCl, 0.01% gelatine), and 15.3 μL of nuclease free water) was added to 4 μL of DNA. The primary amplification was carried out under the following conditions: 94 °C for 4 min, 35 cycles at 94 °C for 30 s, 55 °C for 1 min and at 72 °C for 1 min, followed by a final extension at 72 °C for 10 min. In the subsequent secondary amplifications, 5 pmoles of species-specific primers were used: FAL1: 5′-TTA AAC TGG TTT GGG AAA ACC AAA TAT ATT-3′ and FAL2: 5′-ACA CAA TGA ACT CAA TCA TGA CTA CCC GTC-3′ for *P. falciparum*, VIV1: 5′-CGC TTC TAG CTT AAT CCA CAT AAC TGA TAC-3′ and V1V2: 5′-ACT TCC AAG CCG AAG CAA AGA AAG TCC TTA-3′ for *P. vivax*, OVAL1: 5′-ATC TCT TTT GCT ATC TTT TTT TAG TAT TGG AGA- 3′ and OVAL2: 5′-GGA AAA GGA CAC ATT AAT TGT ATC CTA GTG-3′ for *P. ovale*, MAL1: 5′-ATA ACA TAG TTG TAC GTT AAG AAT AAC CGC-3′ and MAL2: 5′-AAA ATT CCC ATG CAT AAA AAA TTA TAC AAA- 3′ for *P. malariae*, Pmk8: 5′-GTT AGC GAG AGC CAC AAA AAA GCG AAT-3′ and Pmkr9: 5′-ACT CAA AGT AAC AAA ATC TTC CGT A-3′ for *P. knowlesi*. For each of the secondary amplifications, 4 μL of the primary amplification product were used in the PCR reaction (as above) to make a total reaction volume of 25 μL. The PCR was carried out under the following conditions: 94 °C for 4 min, 35 cycles at 94 °C for 30 s, 58 °C for 1 min and at 72 °C for 1 min, followed by a final extension at 72 °C for 10 min. PCR products were analyzed by electrophoresis using 2% agarose gel.

### Parasite cryopreservation and thawing

Positive *P. knowlesi* isolates were cryopreserved in Glycerolyte 57 (Baxter, Belgium). Two volume of Glycerolyte 57 to one volume of infected red blood cells was used for parasite freezing^30^. First, 20% of the Glycerolyte 57 volume was added in a drop-wise manner to the infected red cell suspension while continuously swirling the tube to mix the contents. The tube was then left to stand for 5 min at room temperature before the rest of the Glycerolyte 57 was added. This preparation was aliquoted into cryovials and stored in a liquid nitrogen tank.

When ready to be used, a frozen cryovial was removed from the liquid nitrogen tank and allowed to thaw in a 37 °C water bath. Once thawed, the volume of blood in the cryovial was measured and transferred to a 50 ml falcon tube. Using a drop-wise method, 0.2× volume of 12% NaCl was added and the tube left to stand for 5 min at room temperature. Then, 10× volume of 1.6% NaCl was added drop-wise. The tube was then centrifuged (380 × g, 5 min) and the supernatant removed. Next, 10× volume of 0.9% NaCl was added in a drop-wise method followed by another round of centrifugation (380 × g, 5 min). The supernatant was again removed and the infected red blood cells pellet was resuspended in RPMI 1640 media warmed to 37 °C and then used for *in vivo* or *ex vivo* work.

### Animal infection procedures

Four malaria naive macaques (*M. fascicularis*) were used in this study. All monkeys were bred and grown in animal facilities in a malaria-free environment in Vietnam (Nafovanny) and were 2 years old and weighed 2 kg when used. The animals were kept in individual cages and maintained on commercial non-human primate food pellets supplemented with a variety of fresh fruits.

Approximately 4 × 10^6^ thawed *P. knowlesi* UM01 strain parasite suspended in PBS were inoculated into macaque A intravenously. Peripheral blood was obtained on alternate days to monitor the appearance of parasites. Once a parasite was detected by microscopy, blood films were made daily to monitor the evolution of the infection. Blood films were stained with 10% Giemsa. On day eight post-inoculation the parasitaemia reached 2.6% with mainly late trophozoite stages observed. A total of 2 mL of whole blood was drawn from the infected macaque and parasites were allowed to mature *ex vivo* for 15 hours allowing them to develop into schizonts that then burst to release merozoites that invaded fresh red blood cells. This led to a two-fold increase in the parasitaemia and produced the ring stages needed for cryopreservation as described above.

Between one and two months later, the cryopreserved infected macaque blood was used to inoculate three other malaria naive *M. fascicularis* (macaque B, C and D) using the same method as above. When the parasitaemia reached 1% or more, 2–4 mL of blood were drawn either for cryopreservation or for use in *ex-vivo* invasion assays. Infected macaques were treated with 25 mg/kg of oral mefloquine eight days post-inoculation.

### *Ex vivo* parasite development

About 2–4 mL of pre-treatment blood was collected from infected macaques into heparin tube. The packed red blood cells were recovered by centrifugation (380 × g for 5 min) and then resuspended in culture medium (RPMI 1640 medium supplemented with 2.5 g/L D-glucose, 25 mM HEPES and 20% v/v heat inactivated human AB serum) to approximately a 3% haematocrit and cultured at 37 °C in flasks gassed with a mixture of 90% N_2_, 5% O_2_, and 5% CO_2_. Parasite development and multiplication were monitored by microscopic examination of Giemsa stained thin blood film. Asexual development of the UM01 line was monitored at hour 0, 4, 10, 22 and 24 of culture. Enumeration of sexual and asexual stages was determined using parasites that were cultured *ex vivo* for a few days. A1-H.1 parasites that were cultured at a different time with a similar parasitaemia were used as a control. At least 500 infected cells were counted to calculate the gametocyte conversion rate.

### *In vitro* culture of the A1-H.1 strain

A frozen A1-H.1 sample was thawed and its content measured and transferred to a 15 mL Falcon tube where the same volume of 3.5% NaCl was added drop wise. This mixture was then centrifuged at 380 × g for 5 min and the supernatant discarded. The same volume of 3.5% NaCl were added again and the sample centrifuged as above and the supernatant discarded. This step was repeated one more time. The final pellet was re-suspended to a haematocrit of 2% in pre-warmed modified RPMI 1640 media supplemented with 10% horse serum as described^15^. The suspension was cultured at 37 °C in flasks gassed with a mixture of 90% N_2_, 5% O_2_ and 5% CO_2_. The media was changed every other day. Parasite multiplication and developmental stages were monitored by examination of Giemsa-stained thin blood film. Two days prior to the invasion assays, the culture media was changed to RPMI 1640 medium supplemented with 2.5 g/L D-glucose, 25 mM HEPES and 20% *v/v* heat inactivated human AB serum, similar to that used in the *ex vivo* parasite development as described above.

### Schizont purification

The MACS magnetic separation technique was used to purify schizonts. The MACS® (25 LD colums, Miltenyi Biotec, Germany) columns, held in a Quadro MACS® magnetic support were filled with pre-warmed (37 °C) RPMI 1640 media. Blood from *ex-vivo P. knowlesi* culture or from infected macaques was diluted with RPMI 1640 media to achieve 50% haematocrit and deposited on the top of the MACS® column. Once blood has gone through the column, media was further added until the eluent became free of red blood cells. The column was then removed from the magnetic field and 2 mL of media to elute the schizonts. The eluent was centrifuged (380 × g, 10 min) and the schizont-rich pellet was used for the invasion assays. The parasitaemia was determined by microscopic examination of Giemsa-stained smears.

### Blood preparation and reticulocytes enrichment

Five mL of blood from healthy type O human volunteers or from healthy macaques were collected by venous puncture into lithium-heparinised tubes. ABO blood typing was done using anti-A and anti-B (Bio-Rad, USA) reagent according to the manufacturer’s protocol. For Duffy antigen typing, anti-Fy^a^ and anti-Fy^b^ sera (Lorne Laboratories) were used. The plasma was removed and the remaining blood fraction was washed three times with RPMI 1640 media and finally adjusted to 50% haematocrit using RPMI 1640 and kept in 4 °C. The blood was used for the invasion assay within 1 month of preparation.

For reticulocytes enrichment, packed red blood cells were washed in RPMI 1640 medium and the white blood cells and platelets were depleted using 2 rounds of CF11 (Whatman) column filtration^28^. The recovered packed red cells were then adjusted to a 50% haematocrit using RPMI 1640 medium, and the mixture was split into 5-mL aliquots that were each carefully layered on a 6 mL 70% Isotonic Percoll cushion. After centrifugation for 15 minutes at 1200 *g*, the resulting fine band of concentrated reticulocytes formed on the Percoll interface was carefully removed and washed twice in RPMI 1640 medium. The washed and concentrated reticulocyte preparations were kept at 4 °C in RPMI 1640 medium at a 20% haematocrit, and were used for the invasion assays within 1 month of preparation. Before use, the proportion of reticulocytes (containing reticular matter) was determined by supravital staining with new methylene blue.

### Antibodies

The MAb FY6, which recognizes the 2C3 epitope on the DARC N-terminal region located on the RBC surface membrane, was generously donated by Professors Yves Colin Aronovicz and Olivier S. Bertrand (Université Paris Diderot, France). Anti-Fy^b^ antibody (EP2546Y) was purchased from Abcam (USA).

### Invasion assay

Purified schizont preparation was mixed with target red blood cells (i.e. human normocytes or reticulocytes, macaque normocytes or reticulocytes) so that the starting schizont parasitaemia was no more than 12%. The mixture was diluted to 4% haematocrit using complete RPMI 1640 media, and different aliquots prepared. The Fy6 Mab or anti Fyb antibody were then added to one of the aliquots (or not for the control), at a final concentration of 25 μg/mL and 20 μg/mL, respectively. 100 μL aliquots of each mixture were then distributed in a 96-well plate and gassed with 90% N_2_, 5% O_2_, and 5% CO_2_. The cultures were then incubated in an incubator at 37.5 °C for an average of 15 hours, which may be extended to 20 hours depending on the stage of parasite maturation, to allow re-invasion to occur. Technical replicates were made for each experiment when sufficient quantities of schizont material were available. Thin blood smears were made from each well at the end of the incubation period and the number of rings/trophozoites in 4000 erythrocytes was counted by examining the Giemsa stained thin smears under light microscope.

### Statistical analysis

1-way ANOVA and Tukey’s Multiple Comparison Tests were performed using GraphPad Prism version 5.00 for Windows, GraphPad Software, San Diego California USA.

## Additional Information

**How to cite this article**: Amir, A. *et al.* Invasion characteristics of a *Plasmodium knowlesi* line newly isolated from a human. *Sci. Rep.* **6**, 24623; doi: 10.1038/srep24623 (2016).

## Supplementary Material

Supplementary Information

## Figures and Tables

**Table 1:** *P. knowlesi* (UM01 and A1-H.1 strains) asexual and sexual stages parasitaemia values with gametocyte conversion rate from *ex vivo*/*in vitro* culture in macaque normocytes.

*P. knowlesi* strain	*Ex vivo/In vitro* culture	Parasitemia (%)		Gametocyte conversion rate
		Asexual stages	Sexual stages	
UM01	Day 1	1.1	0.07	5.5
	Day 2	5.3	0.06	1.1
	Day 3	6.6	0.06	0.9
	Day 4	8.9	0.03	0.4
	Mean ± SD	5.5 ± 2.8	0.06 ± 0.02	2.0 ± 2.4
A1-H.1	Day 1	1.0	0	0
	Day 2	1.9	0	0
	Day 3	9.2	0	0
	Day 4	10	0	0
	Mean ± SD	5.5 ± 4.7	0	0
