# Supplementary material for: Invasion characteristics of a Plasmodium knowlesi line newly isolated from a human
Source: Sci Rep. 2016 Apr 21;6:24623. doi: 10.1038/srep24623 (PMC4838912; doi:10.1038/srep24623)
Supplement: Supplementary Information [file srep24623-s1.pdf]

Invasion characteristics of a *Plasmodium knowlesi* line newly isolated from a human

Amirah Amir[1], Bruce Russell[2], Jonathan Wee Kent Liew[1], Robert W. Moon[3], Mun Yik Fong[1], Indra Vythilingam[1], Vellayan Subramaniam[6], Georges Snounou[4, 5*] & Yee Ling Lau[1*]

Supplementary information

Table S1: ***P. knowlesi*** **(UM01 and A1-H.1 strains) invasion parasitaemia values in human and macaque, normocytes and reticulocytes. Numbers in brackets are normalized parasitaemia values.**

Experiment	Parasitaemia (%)				
	Hour 0	Post reinvasion (Hour 15-20)			
		Human normocytes	Human reticulocytes	Macaque normocytes	Macaque reticulocytes
**1 (UM01)**	8.9	10.0 (1.1)	29.3 (3.2)	15.1 (1.7)	31.8 (3.5)
			14.8 (1.7)	10.6 (1.2)	27.2 (3.1)
				14.0 (1.6)	21.6 (2.4)
**2 (UM01)**	11.3	21.8 (1.9)	29.3 (2.6)	25.8 (2.3)	31.2 (2.8)
				18.4 (1.6)	34.6 (3.1)
**3 (UM01)**	1.9	4.1 (2.2)	18.0 (9.5)	8.1 (4.3)	
		4.2 (2.2)		10.2 (5.4)	
		4.1 (2.2)		9.2 (4.8)	
		3.2 (1.7)			
**1 (A1-H.1)**	0.5	1.1 (2.2)	4.5 (9.0)	1.7 (3.4)	
		1.1 (2.2)	4.3 (8.6)	1.3 (2.6)	
		1.0 (2.0)		1.7 (3.4)	
**2 (A1-H.1)**	1.1	2.8 (2.5)	8.3 (7.5)		
**4 (A1-H.1)**	2.8			7.8 (2.8)	14.0 (5.0)
				11.1 (4.0)	14.2 (5.1)
				11.2 (4.0)	15.6 (5.6)
**5 (A1-H.1)**	3.6			10.1 (2.8)	17.9 (5.0)
				11.2 (3.1)	16.3 (4.5)
				11.5 (3.2)	12.6 (3.5)

Table S2: ***P. knowlesi*** **(UM01 and A1-H.1 strains) invasion parasitaemia values in human or macaque normocytes and in the presence of MAB Fy6 and anti-Fy^b. Numbers in brackets are percentage inhibition values.**

Experiment	Parasitaemia (%)						
	Human normocytes			Macaque normocytes			Duffy negative human normocytes
	Control	Anti-Fy6	Anti-Fy^b	Control	Anti-Fy6	Anti-Fy^b	
**1 (UM01)**	4.1	0.52 (87.3)	3.7 (9.8)	9.2	9.4 (0)	8.1 (12.0)	0.69 (83.2)
							1.19 (71.0)
							1.38 (66.3)
**2 (UM01)**	5.3	1.0 (81.1)	6.7 (0)	13.2	11.6 (12.1)	10.3 (22.0)	0.7 (86.8)
		0.9 (83.0)	7.7 (0)		9.0 (31.8)		0.8 (84.9)
		1.1 (79.3)			11.0 (16.7)		0.4 (92.45)
**3 (UM01)**	21.8	0.68 (96.9)	22.19 (0)	33.4	26.1 (21.9)	26.5 (20.6)	
**4 (UM01)**				25.6	23.9 (6.6)	6.3 (75.3)	
**5 (UM01)**				2.8	1.9 (32.1)	0.36 (87.1)	
**6 (UM01)**				0.3	0.2 (33.3)	0.04 (86.7)	
**1 (A1-H.1)**	1.1	0 (100.0)	0.7 (36.4)	1.6	1.8 (0)	1.7 (0)	0.02 (98.2)
		0(100.0)					0.03 (97.3)
							0.06 (94.6)
**2 (A1-H.1)**	2.8	0 (100.0)	2.5 (9.3)				0.24 (91.4)
	2.8	0 (100.0)	2.6 (7.1)				
**7 (A1-H.1)**				20.4	15.6 (23.5)	7.7 (62.3)	
**8 (A1-H.1)**				26.3	26.5 (0)	4.4 (83.3)	
